# Incidental neuro-endocrine tumor of the appendix: Case report and literature review

**DOI:** 10.1016/j.amsu.2019.05.015

**Published:** 2019-05-31

**Authors:** Adel Elkbuli, Carol Sanchez, Mark McKenney, Dessy Boneva

**Affiliations:** a Department of Surgery, Kendall Regional Medical Center, Miami, FL, USA; b University of South Florida, Tampa, FL, USA

**Keywords:** Appendix, Appendicitis, Carcinoid tumor, NET G1 (carcinoid), Gastrointestinal neuro-endocrine tumors

## Abstract

**Introduction:**

Neuroendocrine tumors (NETs) are neoplasms that arise from neuroendocrine cells that have properties of both neuronal and endocrine cells. NETs are most common in the small intestine, rectum, and the appendix and frequently termed carcinoid.

**Presentation of case:**

A 30-year-old male presented with abdominal pain and tenderness in the RLQ. Computerized tomography revealed findings consistent with acute appendicitis. The patient underwent an uneventful laparoscopic appendectomy for an acutely inflamed appendicitis. Histopathological examination, showed a 0.5 cm well-differentiated neuroendocrine tumor of the distal appendix, with clear margins. The mitotic rate was <2 mitoses/10 high power field. Following guidelines, no further procedures were performed and follow-up one week later was uneventful.

**Discussion:**

Appendectomy for the treatment of appendiceal NETs smaller than 1 cm has been recommended as the treatment of these neoplasms by the guidelines set by The North American Neuroendocrine Society (NANETS). NANETS recommends right hemicolectomy for tumors originating at the base of the appendix, for tumors >2 cm in size, if there is evidence of lymphovascular or meso-appendiceal invasion, with mesenteric lymph node metastases, or for intermediate or high-grade tumors.

**Conclusion:**

We present the case of a 30-year old male that presented with an appendiceal, well-differentiated NET that manifested as appendicitis and laparoscopic appendectomy was performed. The appendix was resected with clear margins. Given appropriate markers appendectomy can be curative.

## Highlights

• This is a case of acute appendicitis, discovered to have an appendiceal NET on pathology.
• Laparoscopic appendectomy was sufficient for our index case.
• Surgeons need to be aware of different treatment strategies and follow-up surveillance protocols.

## 1 Introduction

Neuroendocrine tumors (NETs) originate from neuroendocrine cells and establish themselves throughout the human body, from the thymus to the pancreas and the GI tract [1]. They are termed neuroendocrine cells because of the properties that make them very similar to both neuronal cells and endocrine cells. For instance, NET cells have dense core granules that are very similar to some neurons. They also share some similarities to endocrine cells because these tumor cells store and secrete monoamines [2]. Historically in 1907, they were considered benign and initially termed “*little carcinomas*” by Ordfer, specifically he termed them “karzinoide” and they were not considered a true neoplasm. The term carcinoid is often used for these NETs that are in the GI tract. With the recent increase in the incidence of these tumors and the increasing number of clinical trials, they have now considered true neoplasms and received the term gastroenteropancreatic NETs because of their immense hetereogeneity [3].

Although NETs are considered rare and usually benign, they are actually more prevalent than are gastric and pancreatic adenocarcinomas [4]. This can partly be attributed to the liberalized use of advanced imaging modalities like Computerized Tomography (CT) [5]. There has been a 70–133% rise in the incidence of appendiceal NET's in the last 10 years [[4], [5], [6]]. Diagnosis of appendiceal NETs is usually established histologically after routine appendectomy and occurs in 0.3–0.9% of appendectomies. Benign appendiceal NETs most commonly affect females and individuals in their early twenties [3,7]. Comparatively, malignant appendiceal NETs have an increased incidence at a mean age of 50 years [[7], [8], [9]]. The prognosis of NET's depends on the stage, grade, primary site, and functionality [10]. Their grade is determined by Ki67 index and/or mitoses/High Power Field (HPF). Grade 1 signifies well differentiated with Ki-67 less than 2% and less than 2 mitoses/10HPF. Grade 2s have a Ki-67 index between 3 and 20% and/or 3–20 mitoses/10HPF. Poorly differentiated NETs are Grade 3 if they are >20% on the Ki-67 index and/or more than 20 mitoses/10HPF and are referred to as neuroendocrine carcinomas whereas well-differentiated neoplasms are termed NETs or “carcinoids.” Studies have shown that the TNM staging is not as helpful as is histopathological features in prognosis for these neoplasms [7].

NETs of the appendix are most often located at the tip, do not commonly affect regional lymph nodes, and uncommonly metastasize to the liver. Most commonly, appendiceal NETs are incidentally diagnosed during appendectomies. If the appendectomy specimen shows a tumor that is greater than 2 cm in size, cross-sectional staging of the abdomen and pelvis is recommended after the operation because size of the neoplasm is associated with metastasis. Larger tumors demonstrate metastatic involvement at diagnosis in one-third of the cases, usually to regional nodes and sometimes to the liver [9]. The challenge for the NETs is that a seemingly common case (appendectomy) on occasion reveals this tumor on pathology, thus a treatment plan must be ready. Also, on a PubMed review using the phrases “Neuroendocrine,” ‘appendicitis” and “case report” only 4 manuscripts in English are encountered over the last 40 years [12,15].

Herein, we present the case of 30-year old male with characteristics of appendicitis and an NET incidentally found during laparoscopy. This case has been reported in line with the SCARE criteria [16].

### 1.1 Presentation of case

**Fig. 1:**
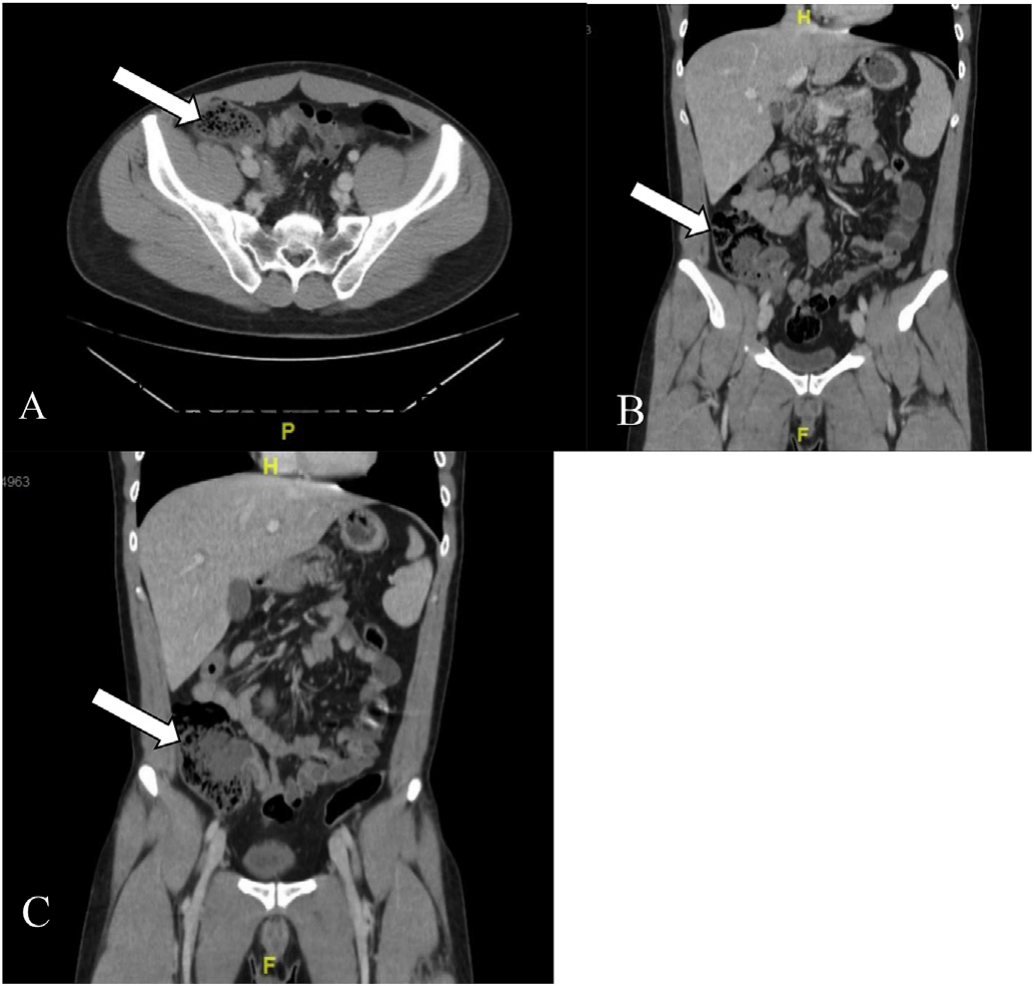
**A**. Acute appendicitis, Right lower quadrant, Abdomen/Pelvis CT scan. **B-** Acute appendicitis, RLQ, CT scan of abdomen/pelvis, coronal view; seen is an appendicolith at the base of appendix. **C-** Acute appendicitis, Right lower quadrant, base of cecum is seen and fluid filled 10–11 mm dilated appendix, CT scan of abdomen/pelvis, Coronal view.

A 30-year-old male presented to the emergency department with a chief complaint of epigastric abdominal pain radiating to the right lower quadrant (RLQ). He felt anorexic, nauseous and had chills and fever. Past medical history was noncontributory. Abdominal examination revealed tenderness in the RLQ with positive Roysing's sign. Laboratory studies indicated a leukocytosis of 15.8K with neutrophilia. CT without oral contrast revealed findings consistent with early acute appendicitis (appendicolith at the appendiceal base with fluid filled appendix measuring up to 10–11 mm (Fig. 1). Informed surgical consent was obtained and he was taken to the operating theater for an uneventful laparoscopic appendectomy for an acutely inflamed, non-perforated appendicitis.

**Fig. 2:**
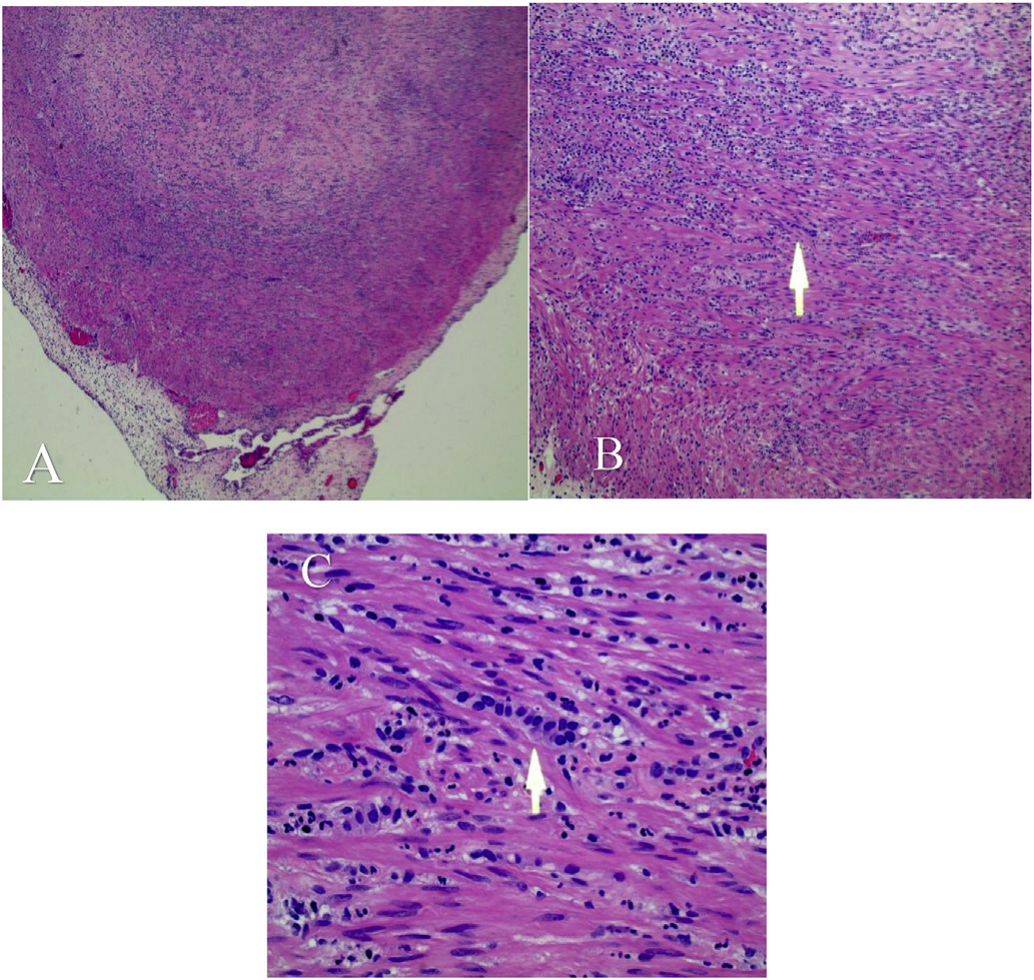
2A, 2B, 2C.Microscopic examination of appendix tip 40 x H&E. Arrow points out cluster of neuroendocrine cells. Inflammatory cells consistent with acute appendicitis are visible.

**Fig. 3:**
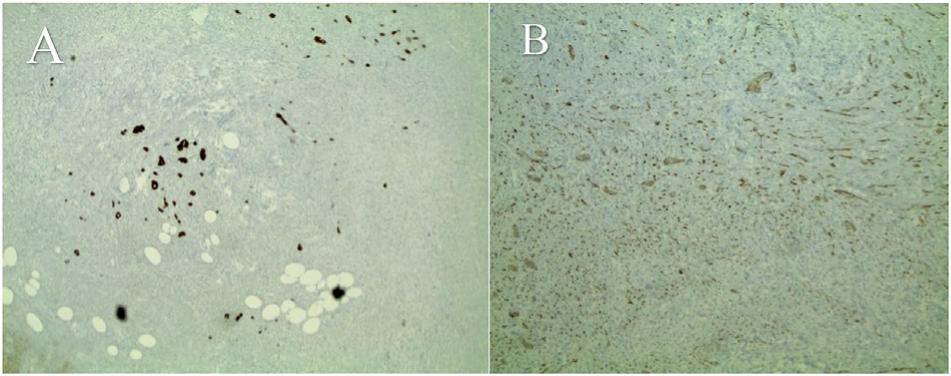
**A**. Positive pankeratin stain in neuroendocrine cells. **B.** Positive Immuno-histochemical stain for synaptophysin 100 x H&E Chromogranin stain was negative.

The patient did well post-up, diet was started and advanced and pain was controlled with oral medications. He was discharged on day 2. His pathology report showed a G1, well differentiated NET at the tip of the appendix invading the muscularis propia up to the serosal surface (Fig. 2). Histology showed acute appendicitis (Fig. 2). Tumor was 0.5 cm in size and a mitotic rate of less than 2 mitoses/10HPF. The Ki67 index was less than 3%. Special histochemical stains performed were positive for pankeratin and synaptophysin (Fig. 3). Margin of resection was free of neoplasm. The patient was doing well on routine follow up in the office post-discharge.

## 2 Discussion

Our patient presented complaining of sharp epigastric pain that started the night before. CT imaging showed evidence of acute appendicitis. The patient underwent an uneventful laparoscopic appendectomy where an incidental G1, well-differentiated NET was discovered on histopathological examination.

Oftentimes NETs of the gastrointestinal tract do not have characteristic symptoms that are unique to its diagnosis. Instead, symptoms often depend on the location of the tumor and the size, presenting symptoms are usually related to the site of the tumor or metastasis. In a population-based study of the outcomes of gastrointestinal NETs, McMullen found that about half of the patients presented with localized disease. The most common presentations for localized disease were suspected appendicitis, abdominal pain with no diagnosis, and GI bleeding or anemia. There has been a large increase in incidence of gastrointestinal NETs, and the elderly population is more susceptible to poorer outcomes in regards to overall and disease-specific survival. Fortunately, for our patient, surgery was both curative and palliative of symptomatology, and is associated with decreased risk of overall and disease-specific death [14].

A review conducted by Amr et al., on 17 cases of NETs revealed that all patients included in their review for appendiceal NETs were suspected of acute appendicitis. In 16 of 17, appendectomy completely resected the tumor, although two patients had to have subsequent right-hemicolectomy because of tumor size being greater than 2 cm. Median follow-up for the patients was 2.9 years, and none of the patients showed evidence of metastases or recurrence. Reaffirming that precise pathologic examination of routine appendectomies is fundamental to the diagnosis [18].

Appendiceal NETs are the most common neoplasm of the appendix, comprising about 32%–57% of all tumors of the appendix, yet the tumors themselves present no specific clinical symptoms [19]. G1 NETs specifically, have an incidence of 0.3–1.1% of routine appendectomies, with some showing no specific visualization on CT or ultrasound imaging [20]. The overall prognosis for NETs of the appendix is good, with tumor size being one of the important determinants of prognosis. According to European guidelines, one of the other determinants of prognosis may also be Ki-67. Studies have shown an association between Ki-67 index and decreased survival [21]. Due to conflicting data, research is warranted. In the present case, the patient's tumor was 0.5 cm in size. For tumors of this size, previous studies have indicated that appendectomy is sufficient. The guidelines set by The North American Neuroendocrine Society (NANETS) reveal that right hemicolectomy is recommended for tumors originating at the base of the appendix, tumors >2 cm in size, if there is evidence of lymphovascular or meso-appendiceal invasion, in patients with lymph node metastases, or for intermediate or high-grade tumors [22,23]. In regards to those tumors between 1 cm and 2 cm, there is no clear consensus. There is uncertainty in this regard due to the lack of studies of such a rare neoplasm, but there have been reports of both lymph node metastases in neoplasms under 1 cm in size that underwent right hemicolectomy, and tumors greater than 2 cm in size but negative lymph nodes [22]. NANETS recommends that for tumors intermediate in size, high-risk characteristics of the neoplasm be taken into account for decision on right hemicolectomy and node dissection [24]. With regards to follow-up on tumors smaller than 1 cm in size, NANETS recommends 3–6 months after resection with curative intent and every 6 months to 1 year for the next 7 years. For tumors that were more advanced, follow-up is advised every 3–6 months and potentially lengthen the interval for patients who show absence of any disease after 12 months [24]. Although NANETS recommends follow-up for tumors <1cm. Murray et al., retrospective study on appendiceal NETs <1 cm indicated that after a 5-year follow up, there were no recurrences or disease-related deaths in individuals affected, which is very similar to previous studies that have shown 0% incidence of nodal metastases in tumors <1 cm. However, their study also sheds light on the variability in follow-up surveillance for patients. Half of the patients in their study did not receive routine surveillance and the other half were referred to medical oncology, imaging, or laboratory studies for surveillance [25]. NET is a rare disease and with reported good prognosis, but it can still be fatal if not handled appropriately. Pathology on appendix specimens should always be complete and follow-up should be done if it's feasible. It is in the best interest of researchers and physicians to raise awareness on this seemingly indolent disease. Further studies are needed on formalized follow-up on these resected NETs along with uniform physician referral and protocols for immunohistochemical analyses in case of recurrent disease.

## 3 Conclusion

We present the case of a 30-year old male that presented with an appendiceal G1, well-differentiated neuroendocrine tumor that manifested as acute appendicitis. A laparoscopic appendectomy was performed. Histopathology report revealed the presence of an appendiceal neuroendocrine tumor at the tip of the appendix with clear margins. This case presents the importance of the increasing incidence of neuroendocrine tumors and the necessity of more studies focusing on the management and long-term implications for these patients with such a rare neoplasm.

## Ethical approval

This case report was conducted in compliance with ethical standards. Informed written consent has been obtained and all identifying information is omitted.

## Author contribution

Adel Elkbuli, Carol Sanchez, Dessy Boneva, Mark McKenney– Conception of study, acquisition of data, analysis and interpretation of data.

Adel Elkbuli, Dessy Boneva, Carol Sanchez - Drafting the article.

Dessy Boneva, Mark McKenney – Management of case.

Adel Elkbuli, Carol Sanchez, Dessy Boneva, Mark McKenney – Critical revision of article and final approval of the version to be submitted.

## Conflicts of interest

None.

## Research registration number

This is a case report study.

## Guarantor

Dessy Boneva.

Mark McKenney.

## Funding

This research did not receive any specific grant from funding agencies in the public, commercial, or not-for-profit sectors.

## Informed consent

Patient informed written consent was obtained and all identifying information is omitted.

## Provenance and peer review

Not commissioned, externally peer reviewed.

## Appendix A Supplementary data

Scare Checklist Scare Checklist
